# Supplementary material for: Data augmentation with improved regularisation and sampling for imbalanced blood cell image classification
Source: Sci Rep. 2022 Oct 27;12:18101. doi: 10.1038/s41598-022-22882-x (PMC9613648; doi:10.1038/s41598-022-22882-x)
Supplement: Supplementary file 1 — Supplementary Information. [file 41598_2022_22882_MOESM1_ESM.pdf]

Data augmentation with improved regularisation and sampling for imbalanced blood cell image classification

Priyanka Rana[1], **Arcot Sowmya**[1], **Erik Meijering**[1], **and Yang Song**[1,*]

[1]School of Computer Science and Engineering, University of New South Wales, Sydney, NSW, Australia.
[*]yang.song1@unsw.edu.au

Supplementary Information

Supplementary Figure S1: Critic loss - epoch training curves for cell-cycle phases dataset.

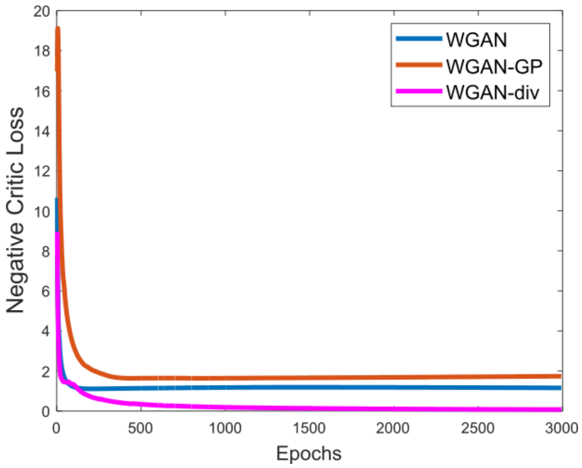

(a) Anaphase.

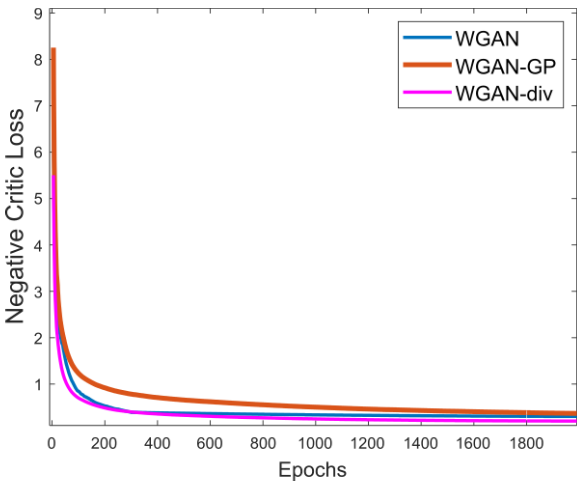

(b) Metaphase.

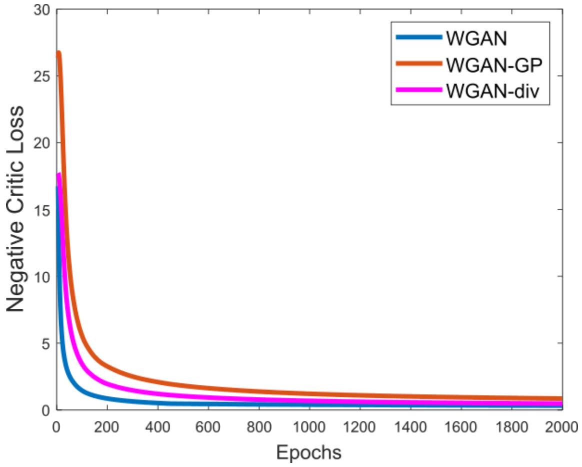

(c) Prophase.

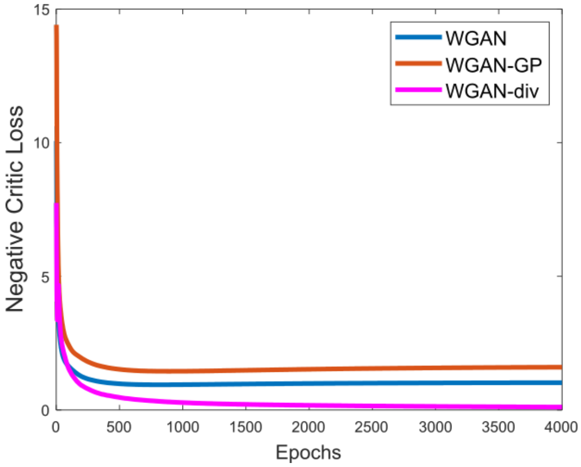

(d) Telophase.

Supplementary Figure S2: Critic loss - epoch training curves for RBC morphologies dataset.

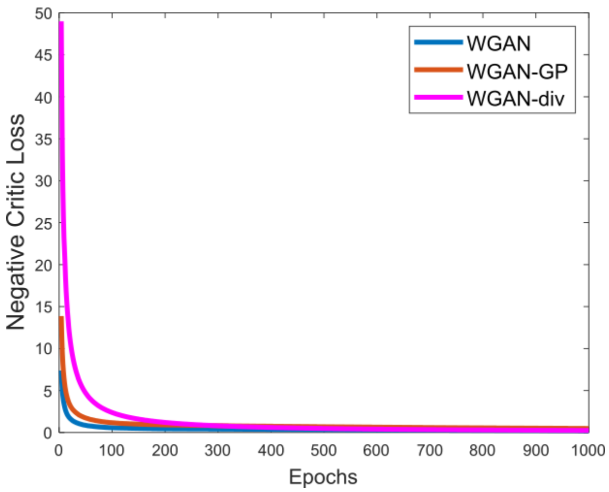

(a) CrenatedDiscoid.

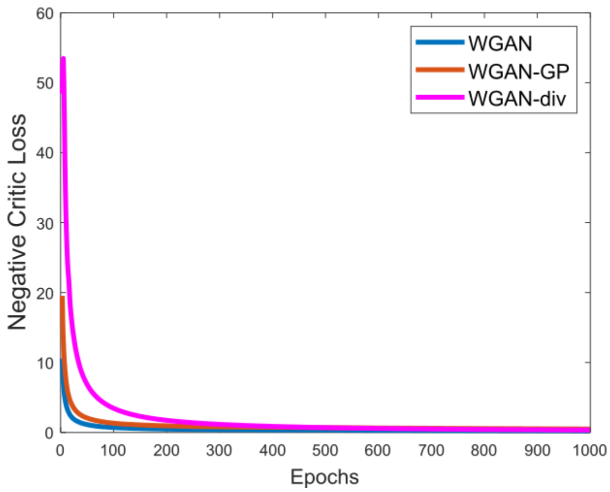

(b) CrenatedSphere.

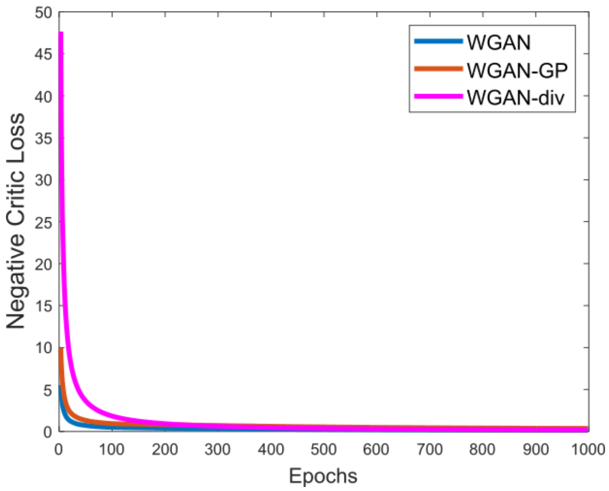

(c) CrenatedSpheroid.

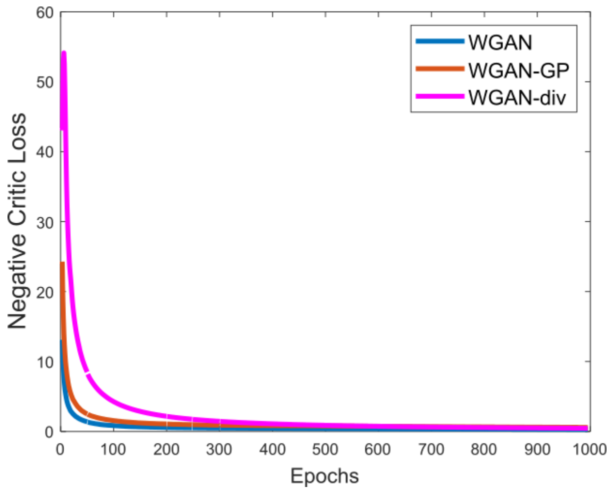

(d) SmoothSphere.

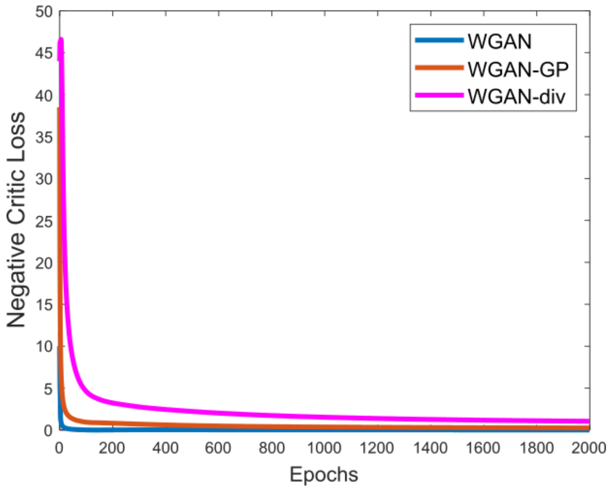

(e) CrenatedDisc.

Supplementary Figure S3: FID Score vs epoch for Cell-cycle phases dataset.

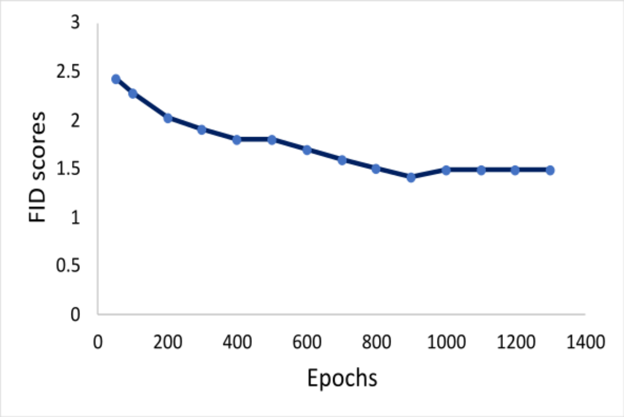

(a) Anaphase.

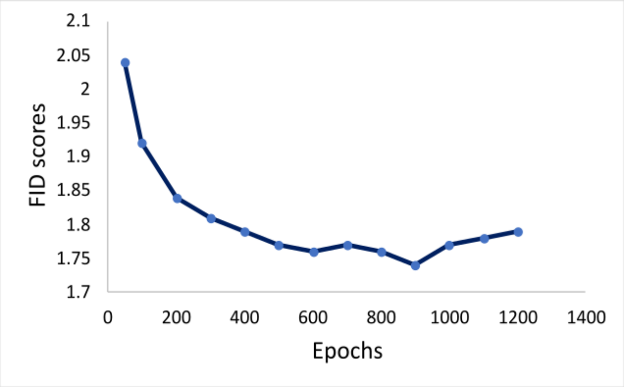

(b) Prophase.

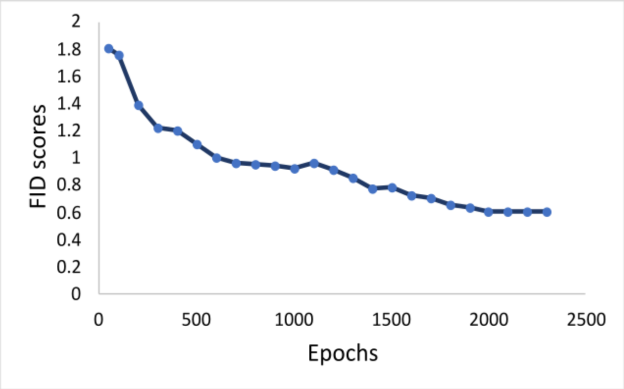

(c) Metaphase.

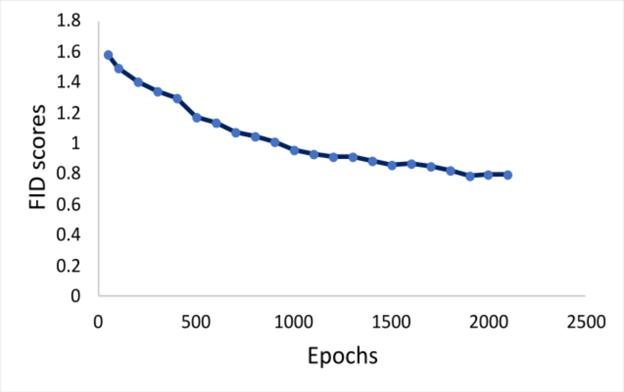

(d) Telophase.

Supplementary Figure S4: FID Score vs epochs for RBC morphologies dataset.

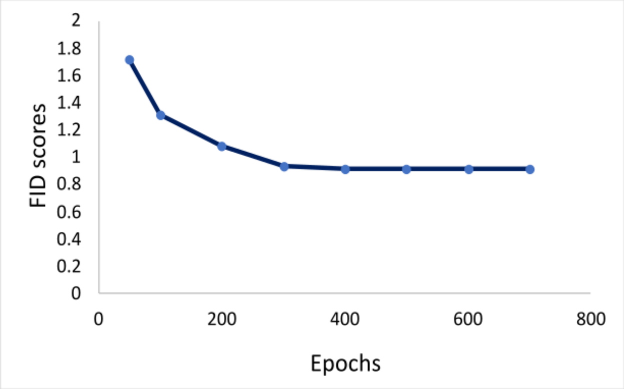

(a) CrenatedDiscoid.

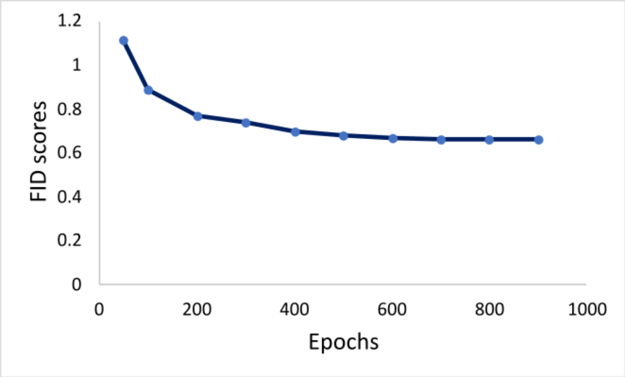

(b) CrenatedSphere.

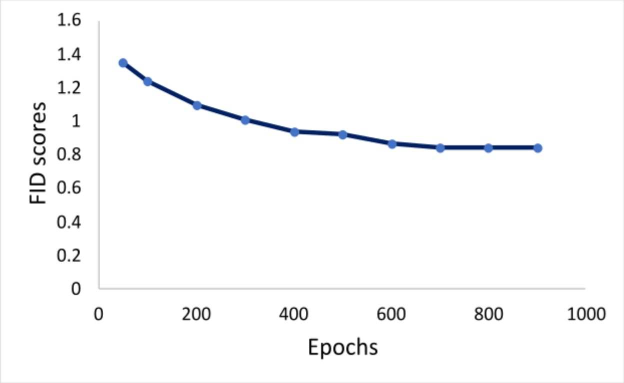

(c) CrenatedSpheroid.

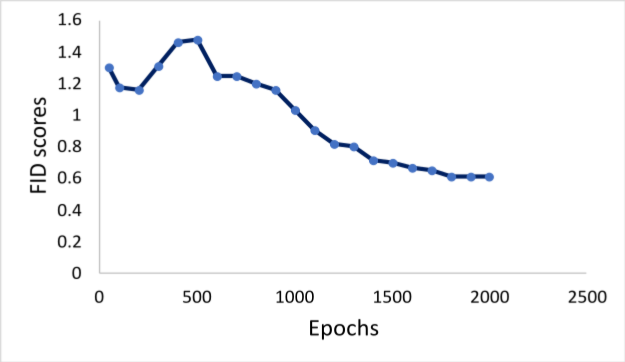

(d) SmoothSphere.

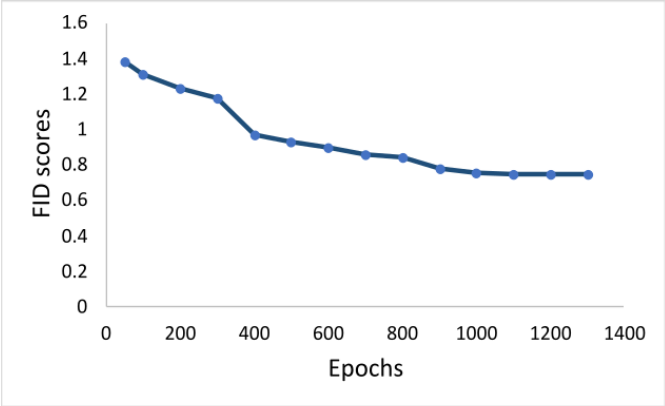

(e) CrenatedDisc.

Cell-Cycle Phases dataset			
**Class**	**NumOfSamples**	**IR**	**n**
G1	14,333	1.0	
S	8,616	1.7	
G2	8,601	1.7	
Prophase	606	23.6	1
Metaphase	68	210.8	2
Telophase	17	843.0	1
Anaphase	15	955.5	2
Average IR		291	

RBC dataset			
**Class**	**NumOfSamples**	**IR**	**n**
SmoothDisc	14,462	1	
Side	9,012	1.6	
CrenatedDisc	5,923	2.4	1
CrenatedSpheroid	4,339	3.3	1
CrenatedDiscoid	3,221	4.5	1
CrenatedSphere	2,187	6.6	1
SmoothSphere	1,808	8.0	1
Average IR		3.9	

Table 1. Imbalance ratio of each class for both datasets.
